# Temporal characterization of the gut microbiome and metabolome in preterm infants

**DOI:** 10.1099/mgen.0.001440

**Published:** 2025-07-30

**Authors:** Lauren C. Beck, Thomas Sproat, Christopher A. Lamb, Andrew Filby, David McDonald, Andrea C. Masi, Gregory R. Young, Sophie Hambleton, Nicholas D. Embleton, Christopher J. Stewart, Janet E. Berrington

**Affiliations:** 1 Translational and Clinical Research Institute, Newcastle University, Newcastle upon Tyne, NE2 4HH, UK; 2 Newcastle Neonatal Service, Newcastle upon Tyne Hospitals NHS Foundation Trust, Newcastle upon Tyne, NE1 4LP, UK; 3 Department of Gastroenterology, Newcastle upon Tyne Hospitals NHS Foundation Trust, Newcastle upon Tyne, NE1 4LP, UK; 4 Biosciences Institute, Newcastle University, Newcastle upon Tyne, NE2 4HH, UK; 5 Great North Children’s Hospital, Newcastle upon Tyne Hospitals NHS Foundation Trust, Newcastle upon Tyne, NE1 4LP, UK; 6 Population Health Sciences Institute, Newcastle University, Newcastle upon Tyne, NE2 4HH, UK

**Keywords:** infant, metabolome, microbiome, preterm

## Abstract

Preterm infants experience abnormal microbial colonization, which coupled with their vulnerable physiology can increase the risk of disease. Understanding the factors influencing the complex relationship between the temporal development of the gut microbiome and functional metabolites derived from microbe–microbe and microbe–host interaction is therefore critical. In this study, 266 longitudinal stool samples from 66 very preterm infants underwent 16S rRNA gene sequencing to analyse gut microbial structure. To further explore the functional status of these gut members, a subset of these samples underwent stool metabolomics (*n*=101). Statistically significant associations were found with age for both the gut microbiota (*P*<0.001) and metabolite profiles (*P*<0.001). Relationships between the gut microbiome and metabolome showed concordance, with 691 significant correlations after adjustment between the top 10 most abundant bacterial taxa and all 977 identified metabolites. *Lactobacillus* had the highest number of significant correlations (31%), amongst which was a strong positive correlation with equol sulphate, an oestrogen produced by intestinal bacteria. This study reveals consistent relationships between the diet, gut microbiota composition and metabolic function. The findings provide valuable insights into the microbial and metabolic dynamics of the preterm gut and the relationships underlying gut microbiome structure and function in vulnerable preterm infants. Further research is needed to confirm these findings and explore their implications for infant health and development.

**Impact Statement**

This study provides critical insights into the relationship between the diet, gut microbiota composition and the metabolome in preterm infants. By identifying correlations between specific bacterial taxa and metabolites, the findings highlight the dynamic microbial and metabolic processes shaping the preterm gut. Further exploration of these interactions will enhance our understanding of early-life health and disease prevention.

## Data Summary

All sequencing data generated and analysed in this study have been deposited in the European Nucleotide Archive under study accession number PRJEB47789. Individual sample accession numbers can be found in supplementary table 8.

## Introduction

The gut microbiome is a dynamic ecosystem involving complex microbe–microbe and microbe–host interactions. Preterm infants display abnormal development of the gut microbiome that is only partially explained by their exposures associated with care in a neonatal intensive care unit (NICU), such as antibiotic use. This abnormal microbial colonization is associated with an increased risk of preterm diseases such as necrotizing enterocolitis (NEC) [1,2] and late-onset sepsis (LOS) [3]. NEC, the leading cause of death in preterm infants after the first week of life, is hypothesized to develop due to a dysregulated immune response to microbial colonization of the gut [4].

Gut microbes produce a wide variety of metabolites, small molecules that serve as the basis of communication with other microbes and host cells [5]. These metabolites are key regulators of gut function and have the potential to modulate host physiology. For example, microbiota-derived short-chain fatty acids such as butyrate have been shown to play essential roles in maintaining gut barrier integrity and supporting gut homeostasis [6]. Other microbiota-derived metabolites including indole derivatives have been shown to influence signalling pathways and potentially contribute to protection against immune-mediated disease [7,9]. Understanding the complex relationships between gut microbes and metabolites is an important and underexplored area of research in very preterm infants (<32-week gestation).

We analysed the longitudinal stool microbiome (*n*=66 infants, *n*=266 samples) and corresponding stool metabolome (*n*=55 infants, *n*=101 samples) of very preterm infants enrolled in a randomized controlled trial (RCT) of exclusive human milk diet. The microbiome dataset has been previously published [10]. Here, we offer a descriptive analysis that integrates the novel metabolome dataset to better understand the microbial and metabolic mechanisms underlying gut development in very preterm infants. This will inform future research into targeted interventions designed to maintain preterm gut health.

## Methods

### Patient cohort and study design

Infants born <32 weeks of gestation were recruited to the ‘Interactions between the diet and gut microbes and metabolism in preterm infants’ (INDIGO; 17/NE/0169) RCT, from four UK NICU sites, with written parental consent covering data and sample collection. The study was approved by Northeast, Tyne and Wear South Research Ethics Committee and was prospectively registered (ISRCTN16799022) [10]. Sixty-six infants were recruited from the Newcastle Royal Victoria Infirmary NICU who form the basis of this report. Demographic information was collected alongside stool samples (*n*=266) at pre-specified time-points. As part of the RCT, infants were randomized to either an exclusive human milk diet (intervention) or standard care (control). The control group consisted of feeding with mothers’ own milk (MOM) and the use of preterm formula milk to make up any shortfall MOM supply. The intervention group consisted of MOM with the use of a ready-to-feed pasteurized human milk product (RTF 26, Prolacta Biosciences, Los Angeles, CA) to make up any shortfall in MOM. We used a breast milk fortifier and aimed to start within 48 h of achieving a milk intake of 150 ml kg^−1^ per day. Infants in the control group received a commercially available, bovine-derived fortifier [Nutriprem (Nutricia Ltd) or SMA Fortifier (SMA Nutrition UK)], and infants in the intervention group received a pasteurized human milk-derived fortifier (P+6, Prolacta Biosciences). Infants were managed with standardized feeding and antibiotic guidance, including the use of probiotics. During the study period, this was the probiotic Labinic™ (*Bifidobacterium bifidum* 0.67×10^9^ c.f.u., *Bifidobacterium longum* subsp. *infantis* 0.67×10^9^ c.f.u. and *Lactobacillus acidophilus* 0.67×10^9^ c.f.u.).

Stool microbiota was analysed by 16S rRNA gene sequencing. Ultra-high performance liquid chromatography (UPLC) MS generated metabolomic data. Samples were selected for analysis as follows: (A) baseline (first sample after enrolment), (B) around day of life (DOL) 10 (anticipated to be before full feeds), (C) at full feeds (150 ml kg^−1^ day^−1^ for 72 h), (D) DOL 21–28 and (E) final sample collected before stopping dietary intervention at 34-week postmenstrual age.

### 16S rRNA gene profiling

DNA was extracted from ~0.1 g of stool using the DNeasy PowerSoil Kit (QIAGEN) following the manufacturer’s protocol with extended bead beating. Sequencing libraries contained kit negatives and sequencing negatives. The V4 region of the 16S rRNA gene was sequenced by NU-OMICS using the 2×250 protocol on the Illumina MiSeq. Raw data processing was performed as previously described [10]. Paired read merging allowed 0 mismatches and a minimum overlap of 50 bases. Merged reads were trimmed at the first base with a *q* less than or equal to 5. Samples were rarefied at 2,000 reads per sample.

### Metabolomic profiling

A subset of stool samples (*n*=101) was also sent for metabolomic profiling at Metabolon (NC, USA). Eighty-five profiles were obtained from the same stool sample that had undergone 16S rRNA gene sequencing, eight were taken on the same day and eight were taken within ±3 days. Samples (~100 mg) were prepared using the automated MicroLab STAR® system from the Hamilton Company. Proteins were precipitated with methanol under vigorous shaking for 2 min (Glen Mills GenoGrinder 2000) followed by centrifugation. The resulting extract was divided into fractions: two for analysis by separate reverse phase (RP)/UPLC-MS/MS methods with positive ion mode electrospray ionization (ESI), one for analysis by RP/UPLC-MS/MS with negative ion mode ESI and one for analysis by hydrophilic interaction liquid chromatography (HILIC)/UPLC-MS/MS with negative ion mode ESI. Samples were placed on a TurboVap® (Zymark) to remove the organic solvent, and sample extracts were stored overnight under nitrogen before preparation for analysis.

All methods utilized a Waters ACQUITY UPLC and a Thermo Scientific Q-Exactive high-resolution/accurate mass spectrometer. The sample extract was dried and then reconstituted in solvents compatible with each of the methods. Each reconstitution solvent contained a series of standards at fixed concentrations. One aliquot was analysed using acidic-positive ion conditions, chromatographically optimized for more hydrophilic compounds. In this method, the extract was gradient eluted from a C18 column (Waters UPLC BEH C18-2.1×100 mm, 1.7 µm) using water and methanol, containing 0.05% perfluoropentanoic acid and 0.1% formic acid. Another aliquot was also analysed using acidic-positive ion conditions; however, it was chromatographically optimized for more hydrophobic compounds. In this method, the extract was gradient eluted from the same C18 column with the addition of acetonitrile and 0.01% FA instead of 0.1% and was operated at an overall higher organic content. Another aliquot was analysed using basic negative ion optimized conditions using a separate dedicated C18 column. The basic extracts were gradient eluted from the column using methanol and water, however with 6.5 mM ammonium bicarbonate at pH 8. The fourth aliquot was analysed via negative ionization following elution from a HILIC column (Waters UPLC BEH Amide 2.1×150 mm, 1.7 µm) using a gradient consisting of water and acetonitrile with 10 mM ammonium formate at pH 10.8. The MS analysis alternated between MS and data-dependent MSn scans using dynamic exclusion. The scan range varied slightly between methods but covered 70–1,000 m/z.

Raw data were extracted, peak identified and QC processed, and biochemical identifications were made using Metabolon’s in-house hardware and software. Peaks were quantified using the area under the curve.

### Clinical variables and statistical analysis

Variables fixed through time (e.g. gestational age at birth, sex and birth mode) are described on a per-infant basis and are constant for all samples for a given infant. Other variables were categorized to reflect exposure in relation to time. The clinical variables used in the analyses were gestational age at birth (continuous), birthweight (continuous), birth mode (vaginal/caesarean), sex (male/female), trial arm (control/intervention), intravenous antibiotics in the past 7 days (no/yes), day of full feed (continuous), NEC requiring surgery or microbiologically confirmed LOS (no/yes), the proportion of enteral feed that is MOM in previous 3 days (continuous; range: 0–100%) and fortifier at time of sample (no/yes).

All statistical analyses were performed in R (https://www.r-project.org/) V.4.0.2, and all visualizations were plotted using the ggplot2 package V.3.3.2 (Wickham, 2016). The Shannon diversity and species richness were calculated using the vegan package V.2.5-7 (https://cran.r-project.org/web/packages/vegan/index.html). The Dirichlet multinomial mixture (DMM) was used to cluster samples on the basis of microbial community structure [11] to determine preterm gut community types (PGCTs). PGCTs were manually ordered youngest (PGCT-1) to oldest (PGCT-5) based on the average DOL of samples within each PGCT. The linear discriminant analysis (LDA) effect size [12] method was used to determine the taxa that discriminated each cluster based on LDA, using default parameters. Microbiome trajectories were manually defined based on five-character strings defining each patient’s movement between PGCTs across the five time-points. To determine which clinical covariates were associated with bacterial and metabolite profiles at each time-point, multiple cross-sectional analyses using the ‘adonis’ function from the vegan package were performed based on the Bray–Curtis dissimilarity. Each test was performed in a stepwise manner, and subsequent *P*-values were adjusted for multiple comparisons using false discovery rate (FDR) adjustment (Benjamini–Hochberg procedure). Linear mixed models and generalized linear mixed models were fit to the data using either the lme4 package V.1.1-29 or the glmmTMB package V.1.0.2.1 (Brooks *et al*., 2017), with subject ID included as a random group intercept. The Multivariate Association with Linear models 2 (MaAsLin2) package V.1.2.032 was used to determine significant taxa and metabolites associated with clinical covariates, whilst adjusting for potential confounders. All covariates used in the ‘adonis’ analysis plus DOL were included as fixed effects in the analysis, and patient identity was included as a random effect. The arcsin square root transformation was performed on relative data, and default MaAsLin2 parameters were used. Features were considered significant with a *Q*-value of <0.05. Spearman’s rank correlation was performed between the 10 most abundant genera and all 977 identified metabolites. *P*-values were adjusted using the FDR method. Regularized canonical correlation analysis (rCCA) was performed using the mixOmics package V.6.31.5. The 50 taxa and metabolites with the highest variance were scaled, and shrinkage parameters were automatically estimated. An empirical *P*-value was calculated via a permutation test with 1,000 permutations.

## Results

Demographic information is presented in Table 1. In brief for the whole cohort, the median gestational age at birth was 27.3 weeks [interquartile range (IQR): 26–28.1] and the median birthweight (IQR) was 912.5 g (675–1110 g). Thirty-six infants were in the control (bovine) arm and 30 were in the intervention (exclusive human) arm with similar demographics (Table S1, available in the online Supplementary Material).

**Table 1.** Patient demographics and sample exposures for the overall cohort (microbiome dataset) and sub-group analysed for metabolomics

	Microbiome	Metabolome
**No. of subjects**	66	55
**No. of samples**	266	101
**Median no. samples per subject (IQR)**	4 (4–5)	2 (2–2)
**Median gestational age (IQR)**	27.4 (26–28.1)	27.6 (26.1–28.1)
**Median birthweight (g) (IQR)**	912.5 (675–1,110)	980 (750–1,145)
**Median day of full feed (IQR)**	13 (11–18)	13 (11–17)
**Median days on antibiotics (IQR)**	11 (6–16.8)	10 (5–16)
**Median days of MOM (IQR)**	46.5 (27.5–66.8)	50 (29.5–66.5)
**Birth mode**		
Caesarean	37 (56.1%)	30 (54.5%)
Vaginal	29 (43.9%)	25 (45.4%)
**Sex**		
Male	36 (54.5%)	32 (58.2%)
Female	30 (45.5%)	23 (41.8%)
**Trial arm**		
Control	36 (54.5%)	30 (54.5%)
Intervention	30 (45.5%)	25 (45.5%)
**NEC**		
No	63 (95.5%)	54 (98.2%)
Yes	3 (4.5%)	1 (1.8%)
**LOS**		
No	57 (86.4%)	49 (89.1%)
Yes	9 (13.6%)	6 (10.9%)
***Antibiotics in the past 7*** * * ***days***		
No	141 (53.0%)	69 (68.3%)
Yes	125 (47.0%)	32 (31.7%)
**Median % enteral MOM in previous 3 days**	100 (37.75–100)	100 (7–100)
**Fortifier at time of sample**		
No	156 (58.6%)	45 (44.6%)
Yes	110 (41.4%)	56 (55.4%)

**Fig. 1.**
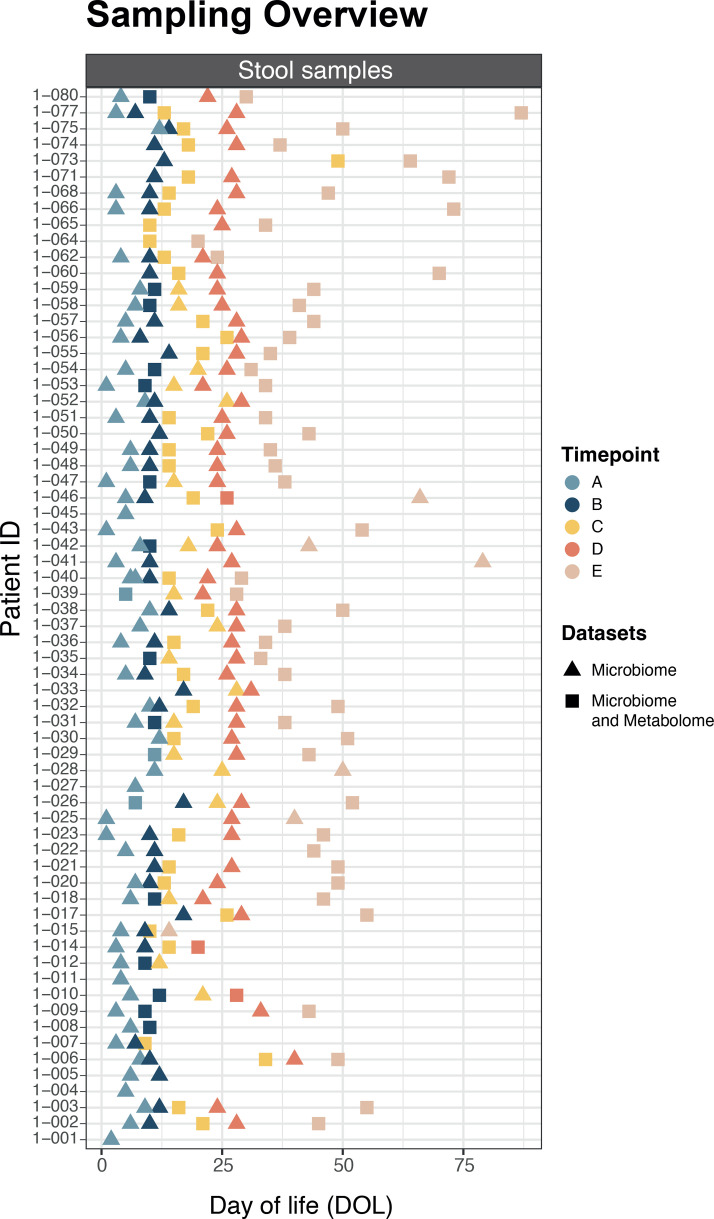
Sampling overview. Samples used in the study from birth to day 90, coloured by pre-defined time-point and shaped based on the dataset(s) the sample was used to generate. Time-points correspond to the following: (A) baseline (first sample after enrolment), (B) around DOL 10 (anticipated to be before ‘full’ feeds), (C) at full feeds, (D) DOL 21–28 and (E) final sample collected before stopping dietary intervention at 34-week postmenstrual age.

Two hundred sixty-six longitudinal stool samples across five pre-defined time-points [A (*n*=55), B (*n*=52), C (*n*=54), D (*n*=52) and E (*n*=53)] underwent 16S rRNA gene sequencing (Table S2). Of these, 101 samples from 55 infants were sent for metabolomics across the 5 time-points [A (*n*=3), B (*n*=14), C (*n*=36), D (*n*=2) and E (*n*=46)] (Fig. 1). Due to the sparsity of samples after DOL 50, all subsequent analysis directly including DOL as a continuous variable was restricted to between DOL 0 and 50 (Fig. 1).

### Stool microbiome profiling

The top five most abundant genera were *Enterobacter/Klebsiella* (cannot be separated based on V4 sequencing; 26.8%), *Bifidobacterium* (18.3%), *Escherichia/Shigella* (cannot be separated based on V4 sequencing; 13.6%), *Staphylococcus* (21.4%) and *Enterococcus* (10.5%). Combined, these five genera accounted for a total of 91% of reads across all samples and a total median relative abundance of 98% (IQR: 89%–100%).

For bacterial profiles, relationships with clinical information were explored by ‘adonis’ analysis. Based on all samples, DOL explained 6.7% of the total variation and corrected gestational age (CGA) explained 6.1% (both *P*<0.001). Categorical time-point explained 10.5% of the total variation (*P*<0.001). Using the first sample from each patient at each time-point, none of the other variables (gestational age at birth, birthweight, birth mode, sex, trial arm, antibiotics in the previous 7 days, day of full feeds, proportion enteral feed that was MOM in previous 3 days, fortifier at the time of sample, NEC or LOS) were significantly associated with overall bacterial profiles at any time-points (Fig. S1).

**Fig. 2.**
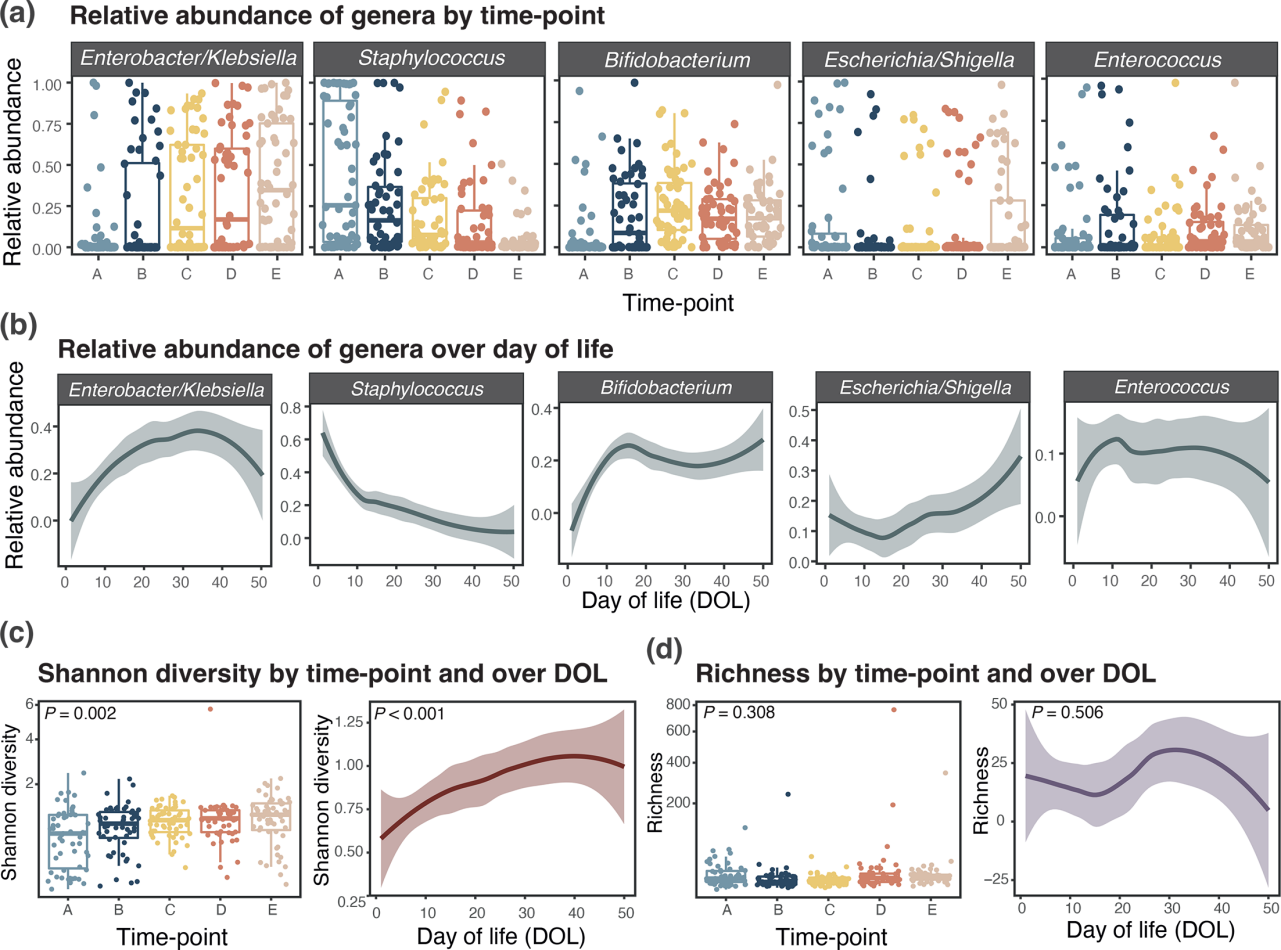
Descriptive overview of the most abundant genera and alpha diversity of the preterm gut microbiome. Relative abundance of the five most abundant genera in this cohort across (**a**) categorical time-points A–E and over (**b**) continuous DOL up to DOL 50. (**c**) Shannon diversity based on OTUs across categorical time-points A–E and over continuous DOL. (**d**) Bacterial OTU richness across categorical time-points A–E and over continuous DOL. Time-points correspond to the following: (A) baseline (first sample after enrolment), (B) around DOL 10 (anticipated to be before ‘full’ feeds), (C) at full feeds, (D) DOL 21–28 and (E) final sample collected before stopping dietary intervention at 34 weeks’ postmenstrual age.

Associations between clinical data and specific bacterial genera were explored by MaAsLin2 analysis. Significant associations were largely attributable to the specific time-points, corroborating the above findings that DOL is the main determinant of the infant gut microbiome (Table S3). The relative abundance of *Enterobacter/Klebsiella* was significantly higher at later time-points D (*P*=0.001, *Q*=0.044) and E (*P*<0.001, *Q*=0.02) compared with the earliest, whilst the opposite was true of *Staphylococcus,* which was most abundant in the first time-point (*P*<0.001, *Q*=0.004) (Fig. 2a). *Bifidobacterium* relative abundance was higher in time-point C (*P*<0.001, *Q*=0.036) compared with A and was observed consistently across all time-points after time-point B, as well as over continuous DOL (Fig. 2a, b). This was likely due to all infants receiving a probiotic product containing *Bifidobacterium*, which commenced when first milk was tolerated (i.e. between time-points A and B). *Veillonella* relative abundance was significantly higher in samples where a fortifier was being given at the time of the sample (*P*=0.001, *Q*=0.043). There was no relationship between either time-point or DOL and the relative abundance of *Escherichia/Shigella* or *Enterococcus*, which remained relatively consistent over time (Fig. 2a, b). The Shannon diversity significantly increased through time-points A to E and based on DOL (both *P*<0.05) (Fig. 2c), but operational taxonomic unit (OTU) richness was not significantly associated with either time-point (*P*=0.309) or DOL (*P*=0.506) (Fig. 2d).

### Bacterial community-type profiling

**Fig. 3.**
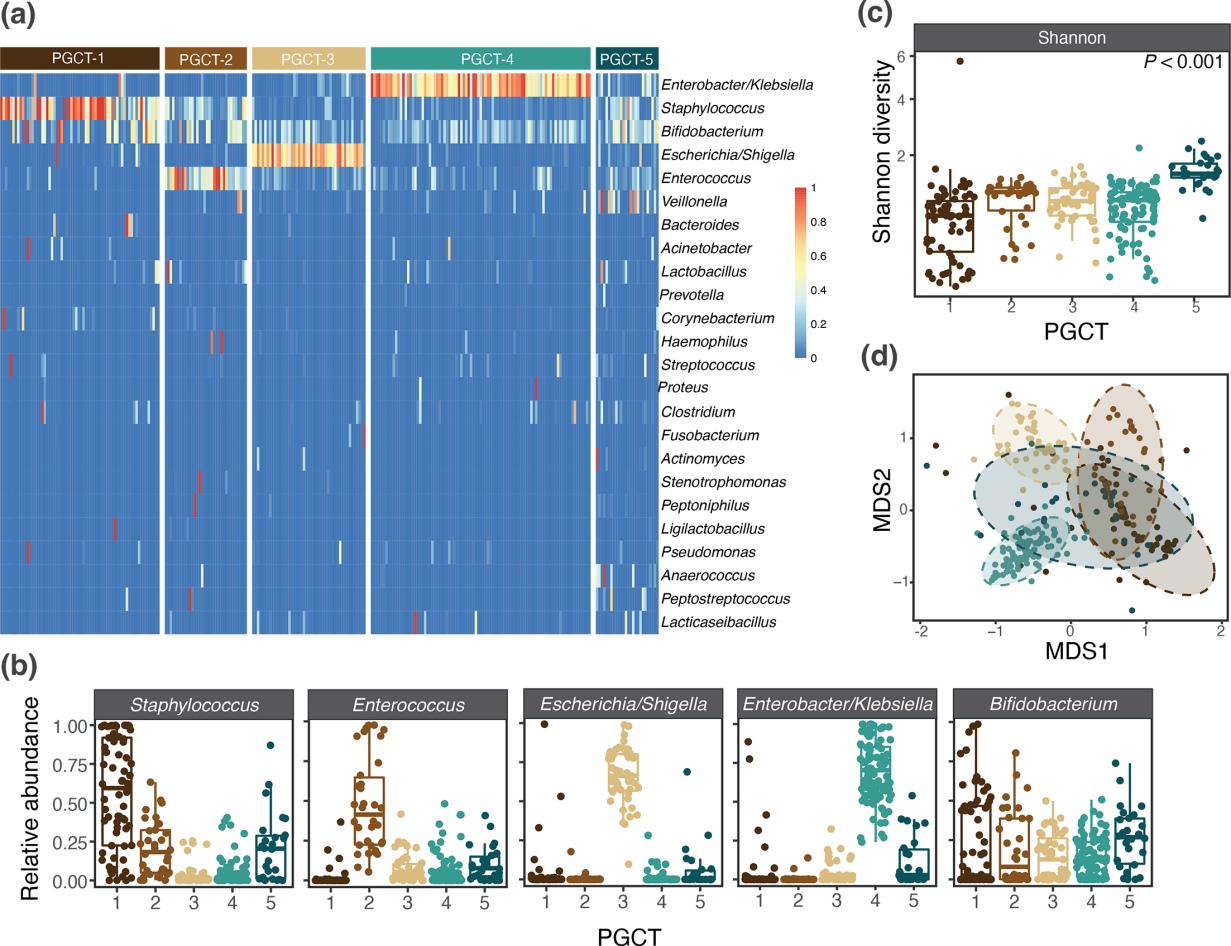
(**a**) Heatmap of all samples showing the relative abundance of the most dominant taxa, stratified by PGCT. (**b**) Relative abundance of the top five most abundant taxa across PGCT. (**c**) Box plot showing the Shannon diversity for each PGCT. The centre line denotes the median, the box limits denote the IQR and the whiskers extend to the limits. (**d**) NMDS ordination based on gut bacterial profiles, with 95% CI ellipses, coloured by PGCT.

DMM modelling of bacterial profiles was used to determine PGCTs, which were ordered 1–5 based on the average age of samples within that cluster. Clinical information corresponding to each PGCT is presented in Table 2. PGCT-1 was discriminated by *Staphylococcus*, PGCT-2 was discriminated by *Enterococcus*, PGCT-3 was discriminated by *Escherichia/Shigella* and PGCT-4 was discriminated by *Enterobacter/Klebsiella* (Fig. 3a, b). There was no single discriminatory feature identified for PGCT-5 (Fig. 3a, b), which was characterized as having a significantly higher Shannon diversity (all adj. *P*<0.001) in comparison with all other PGCTs (Fig. 3c). This lack of dominance by a single taxon is also evident in the non-metric multidimensional scaling (NMDS) ordination, where the 95% confidence interval (CI) ellipsis of PGCT-5 shows large overlap with the other PGCTs (Fig. 3d).

**Table 2.** Patient demographic data and sample exposures corresponding to PGCT

	PGCT-1	PGCT-2	PGCT-3	PGCT-4	PGCT-5
**No. of samples**	68	34	47	91	26
**No. of patients**	40	19	24	40	17
**Median no. of samples per subject**	1 (1–2)	2 (1–2)	1 (1–3)	2 (1–3)	1 (1–2)
**Median DOL**	10 (5.8–14)	14 (10–23.3)	24 (11–32)	24 (14–33.5)	26.5 (11.5–40.3)
**Median gestational age**	27.4 (26.4–28.1)	27 (26.1–28)	27 (25.8–27.9)	27.7 (26.1–28.1)	27.1 (26.4–27.9)
**Median birthweight (g)**	865 (640–1,065)	870 (680–1,070)	947.5 (662.5–1,090)	942.5 (712.5–1,042.5)	990 (860–1,040)
**Median day of full feeds**	13 (11–17.5)	16 (12–20)	12.5 (11–18)	13 (11–18)	12 (11–15)
**Median days on antibiotics**	9.5 (6–18.3)	10 (5.5–16)	13.5 (6–17.3)	11 (5.8–16.5)	9 (5–12)
**Median days of MOM**	46 (26.5–65.5)	58 (29.5–84)	51.5 (37.8–72.5)	53 (29.4–65.3)	61 (35–66)
**Birth mode**					
Caesarean	25 (62.5%)	11 (57.9%)	13 (54.2%)	25 (62.5%)	9 (52.9%)
Vaginal	15 (37.5%)	8 (42.1%)	11 (45.8%)	15 (37.5%)	8 (47.1%)
**Sex**					
Male	21 (52.5%)	10 (52.6%)	13 (54.2%)	23 (57.5%)	8 (47.1%)
Female	19 (47.5%)	9 (47.4%)	11 (45.8%)	17 (42.5%)	9 (52.9%)
**Trial arm**					
Control	23 (57.5%)	13 (68.4%)	15 (62.5%)	24 (60%)	13 (76.5%)
Intervention	17 (42.5%)	6 (31.6%)	9 (37.5%)	16 (40%)	4 (23.5%)
**NEC**					
No	38 (95%)	18 (94.7%)	24 (100%)	38 (95%)	16 (94.1%)
Yes	2 (5%)	1 (5.3%)	0 (0%)	2 (5%)	1 (5.9%)
**LOS**					
No	35 (87.5%)	16 (84.2%)	20 (83.3%)	35 (87.5%)	14 (82.4%)
Yes	5 (12.5%)	3 (15.8%)	4 (16.7%)	5 (12.5%)	3 (17.6%)
**Antibiotics in past 7 days**					
No	23 (33.8%)	18 (52.9%)	28 (59.6%)	56 (61.5%)	16 (61.5%)
Yes	45 (66.2%)	16 (47.1%)	19 (40.4%)	35 (38.5%)	10 (38.5%)
**Median % enteral MOM previous 3 days**	100 (100–100)	100 (0.8–100)	100 (77.5–100)	100 (49.5–100)	100 (28.8–100)
**Fortifier at time of sample**					
No	59 (86.8%)	27 (79.4%)	23 (48.9%)	36 (39.6%)	11 (42.3%)
Yes	9 (13.2%)	7 (20.6%)	24 (51.1%)	55 (60.4%)	15 (57.7%)

Of the 66 infants included in the study, 58 had 3 or more samples across the defined time-points. These infants could be categorized into 1 of 6 microbiome trajectories during this early-life period based on movement between PGCTs across these time-points (Fig. S2). These trajectories are characterized as follows: type 1 (fluctuation between PGCTs), type 2 (stable ‘early’ PGCT), type 3 (stable ‘late’ PGCTs, mostly ‘pathobiont’-dominant), type 4 (shift to *Escherichia/Shigella*-dominant PGCT-3), type 5 (shift to *Enterobacter/Klebsiella*-dominant PGCT-4) and type 6 (shift to PGCT-5). Clinical information corresponding to patients assigned to each trajectory is described in Table S4. The greatest proportion of infants (*n*=16, 27.6%) exhibited a type 5 trajectory and experienced a shift into an *Enterobacter/Klebsiella*-dominant community.

### Stool metabolome profiling

**Fig. 4.**
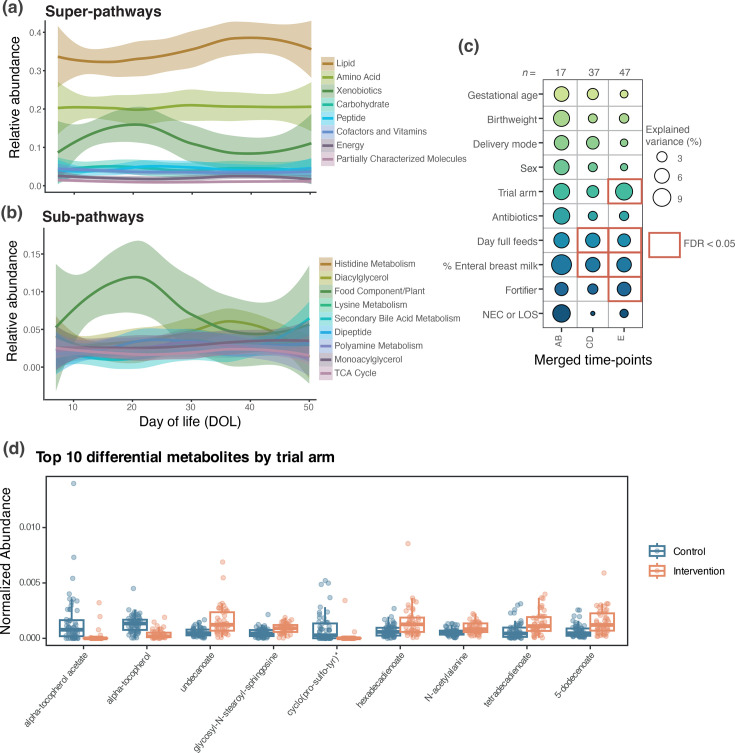
Overview of stool metabolomic profiles. Relative abundance based on locally weighted estimated scatterplot smoothing (LOESS) fits (95% CI) over DOL of (**a**) the most abundant super-pathways excluding unnamed and (**b**) the most abundant sub-pathways excluding unnamed. (**c**) The explained variance of ten clinical covariates at different time-points based on overall stool metabolomic profiles, modelled by ‘adonis’. Bubbles show the explained variance (per cent) by each covariate at a given time-point, and significant results (FDR<0.05) are surrounded by a red box. (**d**) Top ten differential metabolites by trial arm, as identified by MaAsLin2.

In 101 samples from 55 infants (Table 1), a total of 977 unique metabolites were distinguished, of which 833 (85.3%) had confirmed identity using analytical standards. Known metabolites are annotated with a ‘super-pathway’ corresponding to a general metabolic class and a ‘sub-pathway’, which represents more specific metabolic pathways. Based on super-pathways, the majority of identified metabolites were lipids and aa (Fig. 4a). Metabolites remained relatively constant over time at both super-pathway and sub-pathway level, apart from the xenobiotics super-pathway, which increased up to around DOL 20 before decreasing, and the sub-pathway ‘food component/plant’ (i.e. part of the xenobiotics super-pathway), which followed the same trend (Fig. 4a, b).

**Fig. 5.**
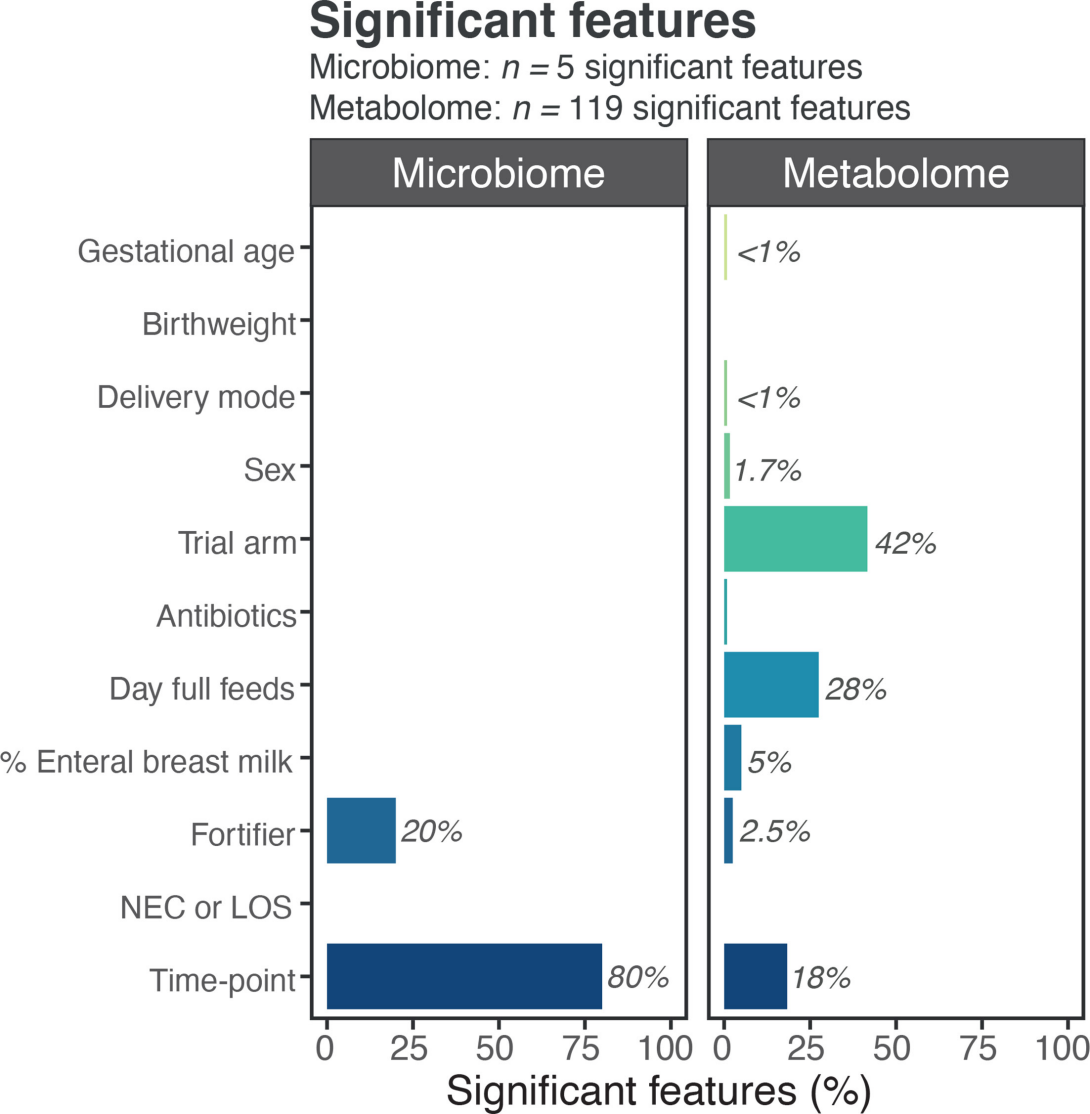
Significant features by clinical variable and unexplained variance across datasets. Percentage of significant features identified by MaAsLin2 analysis attributed to each clinical variable within stool microbiome and metabolome profiles. The majority of significant associations with stool bacterial profiles were attributed to time-point, compared with metabolite profiles where the trial arm had the highest percentage of associations.

Comparable with bacterial profiles, DOL individually explained the greatest overall variation in metabolite profiles (3%) (*P*<0.001), and CGA explained 2.4% (*P*<0.001). Due to a low number of samples in time-points A (*n*=3) and D (*n*=3), time-points A and B were combined, as were C and D, removing duplicates for individual babies. The combined time-points (AB, CD and E) remained significantly associated with overall metabolite profiles (*P*<0.001). Of the clinical variables assessed, % enteral MOM in the previous 3 days and day of full feeds were significantly associated with the metabolome at time-points CD and E (all FDR *P*<0.05), and fortifier and trial arm were also significantly associated with the metabolome at the last time-point (both FDR *P*<0.05) (Fig. 4c).

MaAsLin2 analysis was used to identify metabolic signatures associated with clinical variables. Of the 119 significant features (*Q*<0.05), the majority (42%) were attributable to the trial arm (Figs4d  5), including a number of unidentified metabolites. Alpha-tocopherol and alpha-tocopherol acetate (vitamin E) were amongst the most significantly enriched metabolites in the control trial arm (i.e. bovine formula and fortification) (Fig. 4d, Supplementary Table 6). Day of full feed had the second highest number of significant metabolites (28%) and then time-point (18%). These findings contrast the bacterial profiling results where four of the five significant associations were attributed to time-point (80%). Nine of the 11 covariates returned significantly associated metabolites, compared with only 2 (time-point and fortifier) for bacterial genera (Fig. 5). Since fortifier exposure was significantly associated with bacterial profiles (*Veillonella* was higher during fortifier use; Table S3), the specific metabolites associated with fortifier were further analysed. Three metabolites were significantly associated with fortifier use, all acylcarnitines [myristoylcarnitine (C14), margaroylcarnitine (C17) and 3-hydroxyoleoylcarnitine] and all positively associated (Supplementary Table 6).

### Relationship between stool microbiome and metabolome

#### At the PGCT level

We next integrated the two datasets and found that bacterial taxonomic-derived PGCTs had a significant association with the overall metabolome profiles, explaining 2.35% of the variation in the data whilst controlling for patient identity (*P*=0.038). Of the 82 metabolites significantly associated with PGCTs, the most significant included 3 aa for PGCT-2 (*Enterococcus* dominant), 2 involved in tyrosine metabolism, tyramine O-sulphate and tyramine and one involved in lysine metabolism, 5-hydroxylysine. For PGCT-3 (*Escherichia/Shigella* dominant), there were 42 significantly associated metabolites, including 20 aa, 6 of which were involved in leucine, isoleucine and valine metabolism, and 11 lipids (Table S5). There were 13 metabolites significantly associated with PGCT-4 (*Enterobacter/Klebsiella* dominant), 5 of which were lipids including lysophospholipids and phosphatidylethanolamines. Lastly, there were 24 metabolites significantly associated with PGCT-5 (diverse cluster), the majority of which (60%) were unidentified.

#### At the genus level

**Fig. 6.**
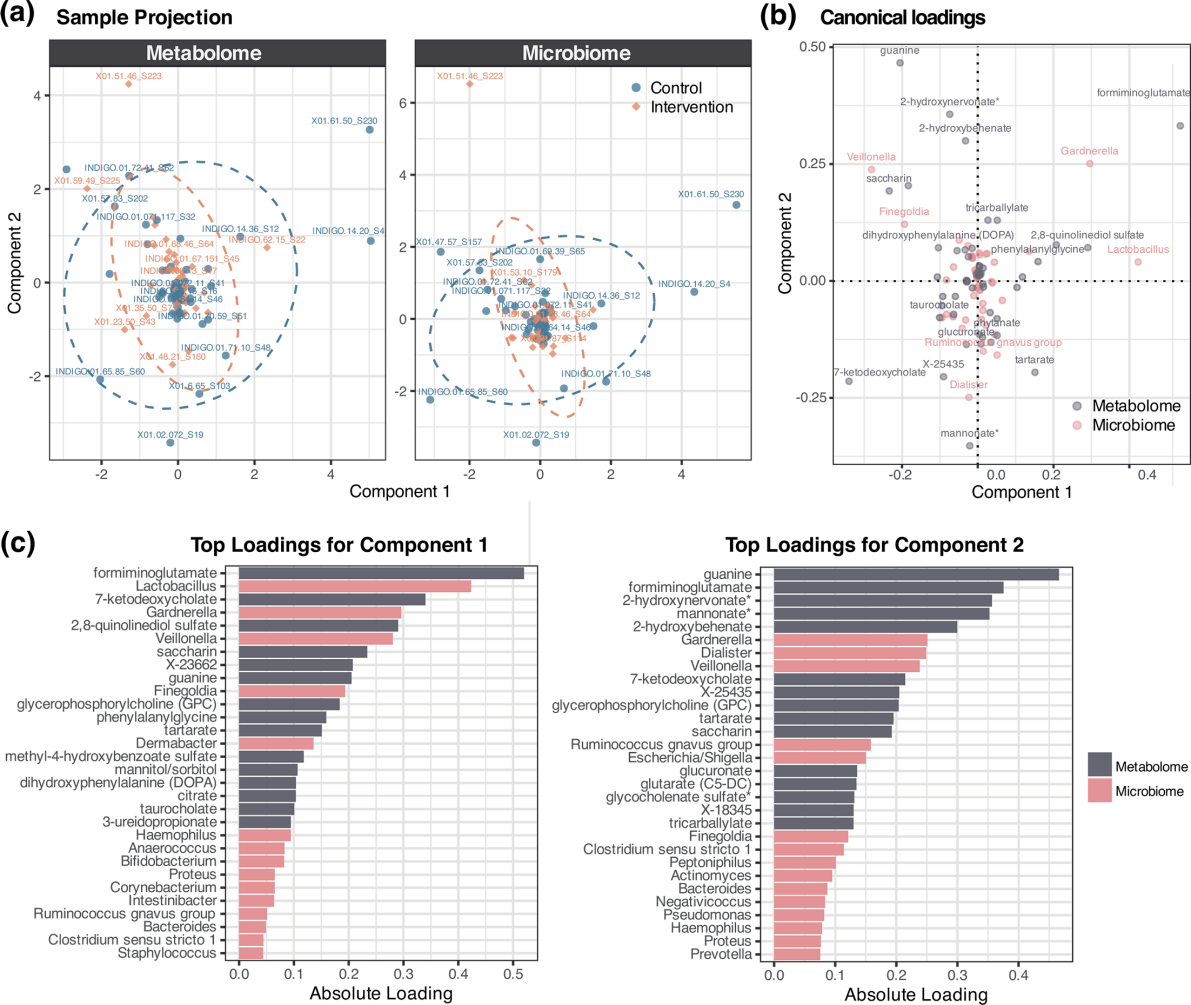
rCCA between the preterm gut microbiome and metabolome. (**a**) Sample projection based on metabolome and microbiome features across components 1 and 2. (**b**) Canonical loadings of microbial and metabolite features across components 1 and 2. (**c**) Top contributing microbial and metabolite features for components 1 and 2.

rCCA was performed to evaluate the multivariate relationship between microbial genus-level relative abundance and metabolomic profiles, based on the 50 genera and metabolites with the highest variance. An empirical permutation test revealed that the canonical correlations for the first 49 components were statistically significant (*P*<0.001; Fig. 6), indicating a shared structure between the datasets. Whilst the results further support the presence of a robust multivariate relationship between the two omic layers, as observed at the PGCT level, the trial arm did not appear to have an impact on the overall structure of both of these datasets (Fig. 6a, b). Formiminoglutamate was found to be the top contributing metabolite for component 1 and also ranked highly for component 2. Guanine was the top contributing metabolite for component 2. Amongst the microbial features, *Lactobacillus* was the top contributing genera for component 1, whilst *Gardnerella* was the top contributor for component 2 and second highest for component 1 (Fig. 6c).

Spearman’s rank correlation analyses between the 10 most abundant genera (*Enterobacter/Klebsiella*, *Bifidobacterium*, *Escherichia/Shigella*, *Staphylococcus*, *Enterococcus*, *Veillonella*, *Lactobacillus*, *Bacteroides*, *Acinetobacter* and *Ligilactobacillus*) and all the 977 metabolites revealed 691 significant relationships after FDR adjustment (402 positive and 289 negative; Supplementary Table 7 and Fig. S3). The highest number of significant correlations was attributable to *Lactobacillus* (31%), identified in the rCCA as being one of the top loadings for component 1, the most significant of which were three xenobiotics (equol sulphate, ferulic acid 4-sulphate and enterolactone sulphate), which were all positively correlated to *Lactobacillus* relative abundance. *Staphylococcus* also had a high number of significant positive and negative correlations (26%). The three most significant were negative correlations with phospholipids, including a lysophospholipid and phosphatidylethanolamine (all *P*<0.001). In contrast, these metabolites were significantly positively correlated to *Enterobacter/Klebsiella* relative abundance and correspondingly were all also significantly enriched in PGCT-4 (*Enterobacter/Klebsiella* dominant). There were 37 metabolites that were significantly correlated with *Veillonella* relative abundance, with a positive correlation being identified with 1 of the acylcarnitines significantly associated with fortifier use (Supplementary Table 6, Supplementary Table 7).

## Discussion

This longitudinal study of preterm infants characterized the relationship between the gut microbiome and metabolome. We extended the previous analysis looking specifically at the impact of an exclusive human milk diet on the microbiome [10], by incorporating stool metabolomics. Our findings highlight that postnatal age had the biggest impact on both the gut microbiome and metabolome. Overall, there was a strong concordance between bacterial taxa and metabolites detected in stool, with >650 significant correlations identified between the most abundant bacterial taxa and metabolites.

Notably, DOL explained more variation in both datasets than CGA. This is consistent with previous research, where the main drivers of gut microbiome development in healthy preterm infants were postnatal age and probiotic use [13], the latter of which is ‘global’ in our study population. At the functional metabolite level, significant associations were found with multiple feeding-related covariates including % enteral MOM in previous 3 days, day of full feeds, fortifier at the time of sample and trial arm (bovine vs. exclusive human diet).

Exploring individual features (i.e. specific bacterial genera or metabolites), fortifier use was the only clinical covariate significantly associated with any bacteria. The use of any fortifier was significantly associated with an increase in the relative abundance of *Veillonella*, as well as the abundance of three specific acylcarnitines. Indeed, one acylcarnitine was directly correlated with the relative abundance of *Veillonella*, as well as five additional acylcarnitines, an association which has been previously described [14]. Whilst the impact of bovine vs. human milk-based fortifiers on the gut microbiome has received recent attention [15,16], the impact of fortification in general on the preterm gut microbiome has not been widely explored. One prospective cohort study noted a decrease in the relative abundance of *Veillonella* in preterm infants receiving a human milk-based fortifier alongside human breast milk compared with infants receiving a bovine milk-based fortifier alongside human breast milk [17].

Whilst the trial arm did not appear to have a large impact on the overall structure of both the microbiome and metabolome datasets, the majority of the 120 significant metabolome associations with clinical variables were related to the trial arm. It is likely that these differences in metabolites, including the most significantly associated (vitamin E), are attributable to the presence or absence of bovine milk-derived products and therefore different direct sources of metabolites in the gut. Vitamin E contents of bovine and human fortifiers are known to differ, and this direct provision of metabolites likely contributed to the observed differences. Other notable metabolites that were differentially abundant between trial arms included a number of lipids, specifically unsaturated fatty acids such as undecanoate, hexadecadienoate, tetradecadienoate and 5-dodecenoate higher in the intervention arm. Whilst we cannot be certain of the source, these differences may reflect the altered transformation of dietary lipids and other components by members of the gut microbiome. The observed shifts in specific stool metabolites suggest that the nutritional intervention influences gut metabolic outputs either through the direct provision of these compounds or via downstream microbial processing. Although the mechanisms remain unclear and the clinical significance is uncertain, these findings highlight the potential for early nutritional strategies to functionally shape the preterm gut environment.

We show a close correlation between specific bacterial genera and specific metabolites. Whilst we are unable to determine if the associated metabolites were microbially produced, there is anecdotal evidence to suggest some are. For instance, equol is an oestrogen produced by intestinal bacteria and was positively correlated with *Lactobacillus* in its sulphate form, with *Lactobacillus* being a known producer of equol [18]. Various gamma-glutamyl aa were found to be positively associated with the relative abundance of both *Bifidobacterium* and *Lactobacillus*, which have been shown to be produced by *Bifidobacterium breve* and *Bifidobacterium infantis* [19, 20]. Such associations underscore the intricate functional interplay between the gut microbiota and the metabolic environment of the preterm gut. Furthermore, the strong relationship between stool bacterial and metabolite profiles observed here is consistent with previous work in preterm infants, which noted a significant microbial-metabolite association [13].

## Conclusions

DOL was the primary driver of both the gut microbiome and metabolome in very preterm infants over the first 50 days of life. Significant associations were also observed between metabolites and clinical covariates, including trial arm. Given the lack of impact of the trial arm on the microbiota observed here, this likely reflects the direct influence of human and bovine milk on the very preterm infant gut metabolome, or else the downstream microbial processing of the dietary components provided. Overall, the results demonstrate the potential to integrate both microbiome and metabolome datasets from longitudinal preterm infant samples, within the framework of an RCT. Incorporating longitudinal sampling of various types and multi-omic analysis into interventional trials in preterm infants holds promise for mechanistic exploration and adds value when evaluating novel clinical interventions [21].

## Supplementary material

10.1099/mgen.0.001440 Uncited Supplementary Material 1.

10.1099/mgen.0.001440 Uncited Supplementary Material 2.

## Abbreviations

CGA: corrected gestational age
CI: confidence interval
DMM: Dirichlet multinomial mixture
DOL: day of life
ESI: electrospray ionization
FDR: false discovery rate
HILIC: hydrophilic interaction liquid chromatography
IQR: interquartile range
LDA: linear discriminant analysis
LOS: late-onset sepsis
MaAsLin2: Multivariate Association with Linear models 2
MOM: mothers’ own milk
NEC: necrotizing enterocolitis
NICU: neonatal intensive care unit
NMDS: non-metric multidimensional scaling
OTU: operational taxonomic unit
PGCT: preterm gut community type
rCCA: regularized canonical correlation analysis
RCT: randomized controlled trial
RP: reverse phase
UPLC: ultra-high performance liquid chromatography
