# Supplementary material for: Temporal characterization of the gut microbiome and metabolome in preterm infants
Source: Microb Genom. 2025 Jul 30;11(7):001440. doi: 10.1099/mgen.0.001440 (PMC12310240; doi:10.1099/mgen.0.001440)
Supplement: Uncited Supplementary Material 1. [file mgen-11-01440-s001.pdf]

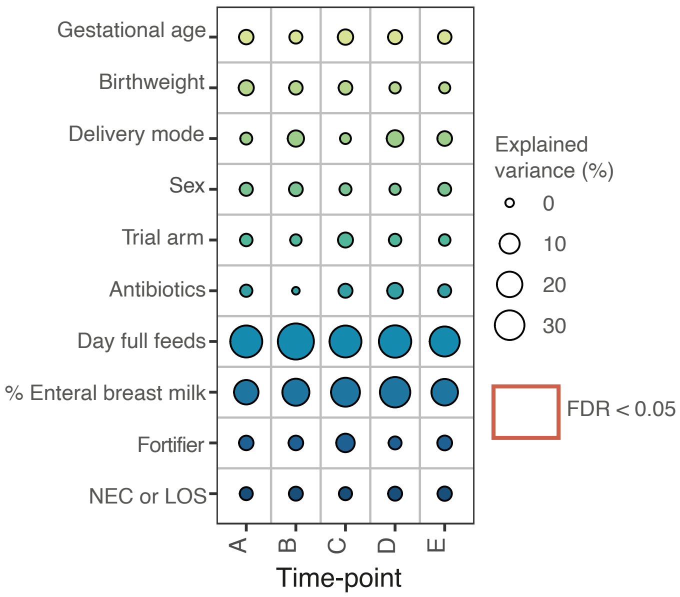

Supplementary Figure 1. The explained variance of 10 clinical covariates at different time-points based on overall stool microbiome profiles, modelled by ‘adonis’. Bubbles show the explained variance (%) by each covariate at a given time-point. No covariates were found to be significant after FDR adjustment.

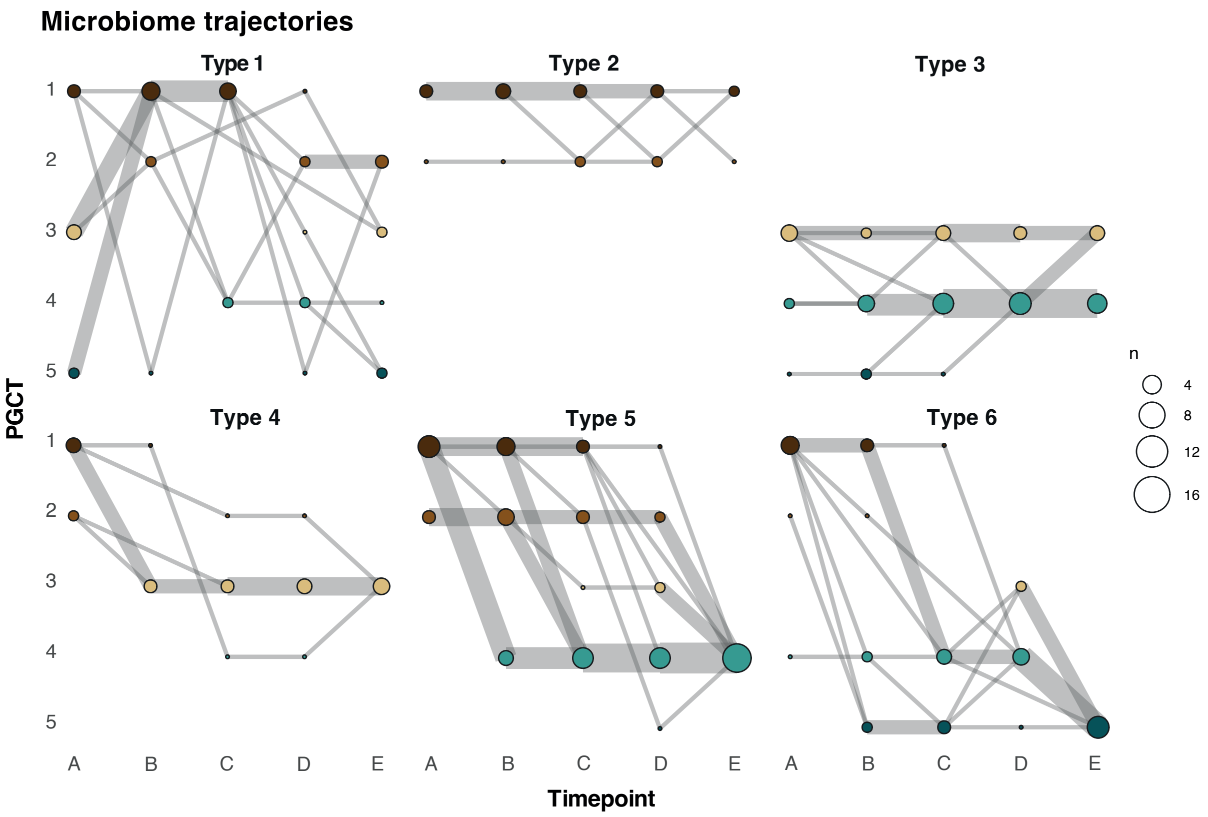

Supplementary Figure 2. Microbiome trajectories through PGCTs across timepoints A-C. 6 microbiome trajectories were defined based on patient movement through PGCTs across timepoints.

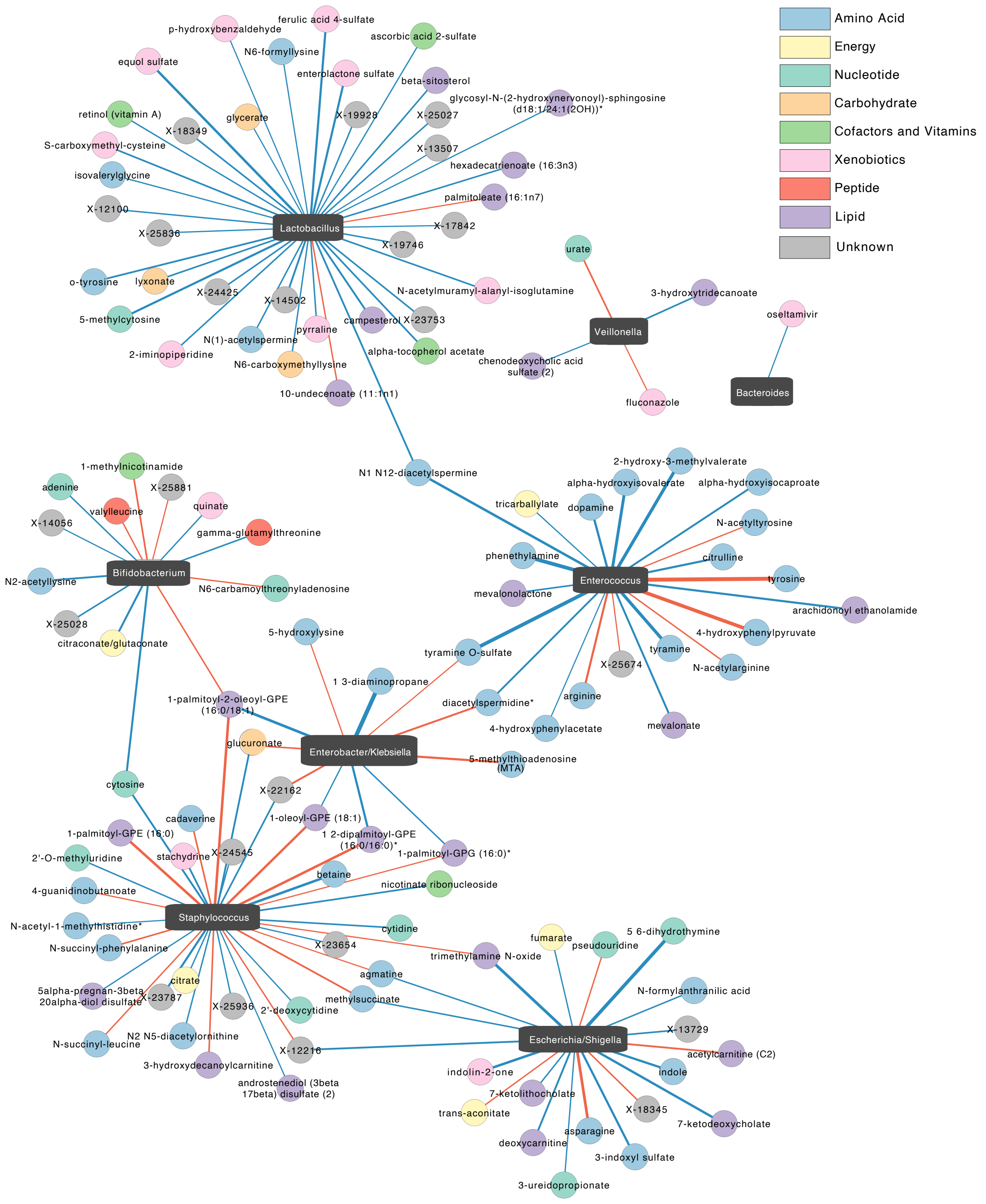

Supplementary Figure 3. Network plot visualising Spearman's rank correlation analysis using a correlation coefficient (R) cutoff of 0.4, between the most abundant taxa and identified metabolites in the preterm gut microbiome. Edges are coloured by positive (blue) or negative (red) interactions and weighted by the correlation coefficient (R). Central nodes denote bacterial taxa and metabolite nodes are coloured by super-pathway.

Supplementary Table 1. Patient demographics of the analytical cohort, stratified by trial arm. *P*-values are based on Chi-squared test for categorical data and Kruskall-Wallis test for continuous data.

	All	*Control*	*Intervention*	*pval*
No. of subjects	66	36	30	
No. of samples	266	152	114	
Median no. samples per subject (IQR)	4 (4 – 5)	5 (4 – 5)	4 (3.25 – 5)	
Median gestational age (IQR)	27.3 (26 – 28.1)	27.1 (26.1 – 28)	27.5 (26 – 28.1)	0.657
Median birthweight (g) (IQR)	912.5 (675 – 1110)	860 (640 – 1008)	1040 (730 – 1155)	0.098
Median day of full feed (IQR)	13 (11 – 18)	13 (11 – 18)	13 (12 – 17.5)	0.479
Median days on antibiotics (IQR)	11 (6 – 16.5)	10 (6 – 18.2)	12.5 (5.25 – 16)	0.949
Median days of MOM (IQR)	46.5 (27.5 – 66.8)	54 (29.2 – 67.5)	42 (26 – 64)	0.395
Birth mode				0.512
Caesarean	37 (56.1%)	22 (61.1%)	15 (50%)	
Vaginal	29 (43.9%)	14 (38.9%)	15 (50%)	
Sex				0.289
Male	36 (54.5%)	17 (47.2%)	19 (63.3%)	
Female	30 (45.5%)	19 (52.8%)	11 (36.7%)	
NEC or LOS				1
No	54 (81.8%)	29 (80.6%)	25 (83.3%)	
Yes	12 (18.2%)	7 (19.4%)	5 (16.7%)	
Antibiotics in the past 7 days				1
No	141 (53.0%)	81 (53.3%)	60 (52.6%)	
Yes	125 (47.0%)	71 (46.7%)	54 (47.4%)	
Median % enteral MOM in previous 3d	100 (37.75 – 100)	100 (93 – 100)	100 (25.8 – 100)	0.069
BMF at time of sample				0.805
No	156 (58.6%)	88 (57.9%)	68 (59.6%)	
Yes	110 (41.4%)	64 (42.1%)	46 (40.4%)	

Supplementary Table 2. Median (IQR) DOL for each of the timepoints where samples were collected.

	Median (IQR) DOL
**Timepoint A**	5 (3.5 – 7)
**Timepoint B**	10 (10 – 11)
**Timepoint C**	16 (14 – 21)
**Timepoint D**	27 (24 – 28)
**Timepoint E**	43 (35 – 50)

Supplementary Table 3. MaAsLin2 results for taxa associated with clinical co-variates in the INDIGO cohort. Mixed-effects linear models using a variance-stabilizing arcsin square root transformation on relative abundance phyla data were used to determine the significance. Patient ID was included as a random effect.

	**Variable level**	Feature	Coeff	Standard error	pval	qval
Trial arm	Intervention	*Lactobacillus*	-0.032	0.014	0.023	0.215
Antibiotics 7d	Yes	*Bifidobacterium*	-0.121	0.039	0.002	0.056
Day of full feeds		*Bifidobacterium*	-0.06	0.021	0.007	0.11
% enteral breast milk		*Veillonella*	-0.029	0.009	0.002	0.055
% enteral breast milk		*Lactobacillus*	-0.021	0.007	0.003	0.073
Fortifier	Yes	*Veillonella*	0.063	0.019	0.001	0.044
Fortifier	Yes	*Staphylococcus*	-0.162	0.07	0.023	0.215
NEC or LOS	Yes	*Bifidobacterium*	0.16	0.059	0.009	0.122
Time-point	E	*Staphylococcus*	-0.401	0.093	<0.001	0.004
Time-point	E	*Enterobacter/Klebsiella*	0.374	0.099	<0.001	0.017
Time-point	C	*Bifidobacterium*	0.233	0.067	0.001	0.036
Time-point	D	*Enterobacter/Klebsiella*	0.307	0.094	0.001	0.044
Time-point	B	*Bifidobacterium*	0.154	0.055	0.006	0.104
Time-point	D	*Staphylococcus*	-0.247	0.089	0.006	0.104
Time-point	E	*Bifidobacterium*	0.175	0.071	0.014	0.182
Time-point	D	*Bifidobacterium*	0.165	0.068	0.016	0.193
Time-point	C	*Enterobacter/Klebsiella*	0.223	0.093	0.017	0.193
Time-point	B	*Escherichia/Shigella*	-0.138	0.059	0.021	0.215

Supplementary Table 4. Patient demographic data corresponding to microbiome trajectory types.

	Type 1	***Type 2***	***Type 3***	***Type 4***	***Type 5***	***Type 6***
No. of patients	9	5	13	6	16	9
Median gestational age	27.3 (26.4 - 28)	28.3 (26.9 - 29.4)	27.7 (26 - 28.1)	26.6 (25.3 - 27.9)	26.9 (25.2 - 28.1)	27.4 (27 - 27.9)
Median birthweight (g)	1,000 (870 - 1,150)	810 (640 - 1,450)	980 (690 - 1,040)	795 (630 - 1,160)	805 (615 - 1,090)	990 (910 - 1,040)
Median day of full feeds	12.0 (11.0 - 15.0)	13.0 (11.0 - 16.0)	12.0 (10.0 - 14.0)	12.0 (11.0 - 24.0)	18.0 (13.0 - 22.0)	11.0 (11.0 - 13.0)
Median days on antibiotics	9 (6 - 13)	12 (8 - 25)	14 (10 - 16)	17 (16 - 18)	12 (6 - 26)	6 (4 - 10)
Birth mode						
Caesarean	4 (44%)	4 (80%)	5 (38%)	4 (67%)	10 (63%)	5 (56%)
Vaginal	5 (56%)	1 (20%)	8 (62%)	2 (33%)	6 (38%)	4 (44%)
Sex						
Female	5 (56%)	2 (40%)	6 (46%)	4 (67%)	7 (44%)	3 (33%)
Male	4 (44%)	3 (60%)	7 (54%)	2 (33%)	9 (56%)	6 (67%)
Trial arm						
Control	7 (78%)	3 (60%)	4 (31%)	3 (50%)	9 (56%)	8 (89%)
Intervention	2 (22%)	2 (40%)	9 (69%)	3 (50%)	7 (44%)	1 (11%)
NEC						
No						
Yes	0 (0%)	0 (0%)	0 (0%)	0 (0%)	2 (13%)	0 (0%)
LOS						
No						
Yes	3 (33%)	1 (20%)	3 (23%)	1 (17%)	0 (0%)	1 (11%)

Supplementary Table 5. MaAsLin2 results for significant metabolites associated with PGCTs. Mixed-effects linear models using a variance-stabilising arcsin square root transformation on metabolite data were used to determine the significance. Patient ID was included as a random effect.

PGCT	Coeff	stderr	pval	qval	Super pathway	Sub pathway	Chemical name
**2**	0.016	0.005	0.001	0.044	Amino Acid	Tyrosine Metabolism	tyramine
**2**	0.018	0.005	0.001	0.033	Amino Acid	Lysine Metabolism	5-hydroxylysine
**2**	0.032	0.007	<0.001	0.003	Amino Acid	Tyrosine Metabolism	tyramine O-sulfate
**3**	0.021	0.004	<0.001	0.002	Amino Acid	Polyamine Metabolism	putrescine
**3**	0.02	0.005	<0.001	0.016	Energy	TCA Cycle	fumarate
**3**	0.009	0.002	0.001	0.033	Amino Acid	Leucine, Isoleucine and Valine Metabolism	isoleucine
**3**	0.009	0.002	<0.001	0.026	Amino Acid	Leucine, Isoleucine and Valine Metabolism	leucine
**3**	-0.017	0.005	<0.001	0.027	Amino Acid	Glycine, Serine and Threonine Metabolism	betaine
**3**	-0.045	0.011	<0.001	0.013	Amino Acid	Alanine and Aspartate Metabolism	asparagine
**3**	0.02	0.004	<0.001	0.005	Amino Acid	Tyrosine Metabolism	tyramine
**3**	0.034	0.009	<0.001	0.03	Amino Acid	Phenylalanine Metabolism	phenethylamine
**3**	0.019	0.005	0.001	0.039	Lipid	Phosphatidylethanolamine (PE)	1-palmitoyl-2-oleoyl-GPE (16:0/18:1)
**3**	0.024	0.005	<0.001	0.005	Amino Acid	Lysine Metabolism	cadaverine
**3**	-0.028	0.007	<0.001	0.011	Nucleotide	Pyrimidine Metabolism, Cytidine containing	2'-deoxycytidine
**3**	-0.05	0.012	<0.001	0.007	Carbohydrate	Aminosugar Metabolism	glucuronate
**3**	0.031	0.008	<0.001	0.015	Amino Acid	Tryptophan Metabolism	3-indoxyl sulfate
**3**	0.015	0.004	0.001	0.032	Lipid	Fatty Acid, Monohydroxy	3-hydroxymyristate
**3**	0.069	0.013	<0.001	0.001	Lipid	Secondary Bile Acid Metabolism	7-ketodeoxycholate
**3**	0.041	0.011	<0.001	0.03	Lipid	Secondary Bile Acid Metabolism	7-ketolithocholate
**3**	0.019	0.004	<0.001	<0.001	Amino Acid	Leucine, Isoleucine and Valine Metabolism	N-acetylisoleucine
**3**	0.015	0.004	<0.001	0.029	Amino Acid	Leucine, Isoleucine and Valine Metabolism	alpha-hydroxyisovalerate
**3**	0.018	0.005	0.001	0.048	Amino Acid	Leucine, Isoleucine and Valine Metabolism	2-hydroxy-3-methylvalerate
**3**	0.026	0.004	<0.001	<0.001	Lipid	Lysophospholipid	1-palmitoyl-GPE (16:0)
**3**	0.027	0.005	<0.001	0.001	Lipid	Lysophospholipid	1-oleoyl-GPE (18:1)
**3**	0.015	0.004	<0.001	0.023	Lipid	Corticosteroids	cortisone 21-sulfate
**3**	0.025	0.006	<0.001	0.005	Cofactors and Vitamins	Pantothenate and CoA Metabolism	pantoate
**3**	0.026	0.007	<0.001	0.016	Lipid	Phospholipid Metabolism	trimethylamine N-oxide
**3**	0.038	0.011	0.001	0.043	Amino Acid	Tyrosine Metabolism	tyrosol
**3**	0.011	0.002	<0.001	0.002	Xenobiotics	Food Component/Plant	indolin-2-one
**3**	0.016	0.003	<0.001	<0.001	Amino Acid	Tryptophan Metabolism	N-formylanthranilic acid
**3**	0.021	0.005	<0.001	0.01	Lipid	Lysophospholipid	1-palmitoyl-GPG (16:0)*
**3**	0.024	0.007	0.001	0.032	Amino Acid	Tyrosine Metabolism	tyramine O-sulfate
**3**	0.03	0.007	<0.001	0.011	Amino Acid	Glutathione Metabolism	2-hydroxybutyrate/2-hydroxyisobutyrate
**3**	0.017	0.005	0.001	0.048	Energy	TCA Cycle	2-methylcitrate/homocitrate

**3**	0.017	0.004	<0.001	0.016	Lipid	Phosphatidylethanolamine (PE)	1,2-dipalmitoyl-GPE (16:0/16:0)*
**3**	0.018	0.005	<0.001	0.027	Amino Acid	Polyamine Metabolism	(N(1) + N(8))-acetylspermidine
**3**	0.013	0.004	0.001	0.031	Lipid	Corticosteroids	11-dehydrocorticosterone sulfate
**3**	0.027	0.007	<0.001	0.019	Amino Acid	Phenylalanine Metabolism	N-succinyl-phenylalanine
**3**	0.029	0.007	<0.001	0.014	Amino Acid	Leucine, Isoleucine and Valine Metabolism	N-succinyl-leucine
**3**	0.066	0.013	<0.001	0.001	Amino Acid	Polyamine Metabolism	N-carbamoylputrescine
**3**	0.063	0.011	<0.001	<0.001	Unknown	Unknown	12216
**3**	0.027	0.005	<0.001	<0.001	Unknown	Unknown	13729
**3**	-0.018	0.004	<0.001	0.002	Unknown	Unknown	22162
**3**	-0.033	0.009	0.001	0.032	Unknown	Unknown	23654
**4**	0.012	0.003	0.001	0.033	Amino Acid	Polyamine Metabolism	1,3-diaminopropane
**4**	0.018	0.005	<0.001	0.02	Lipid	Phosphatidylethanolamine (PE)	1-palmitoyl-2-oleoyl-GPE (16:0/18:1)
**4**	-0.041	0.01	<0.001	0.01	Carbohydrate	Aminosugar Metabolism	glucuronate
**4**	0.012	0.003	<0.001	0.011	Amino Acid	Leucine, Isoleucine and Valine Metabolism	N-acetylisoleucine
**4**	0.017	0.003	<0.001	0.002	Lipid	Lysophospholipid	1-palmitoyl-GPE (16:0)
**4**	0.018	0.005	<0.001	0.014	Lipid	Lysophospholipid	1-oleoyl-GPE (18:1)
**4**	-0.01	0.003	0.001	0.043	Cofactors and Vitamins	Tocopherol Metabolism	gamma-CEHC glucuronide*
**4**	0.022	0.004	<0.001	0.001	Lipid	Lysophospholipid	1-palmitoyl-GPG (16:0)*
**4**	0.015	0.004	<0.001	0.014	Lipid	Phosphatidylethanolamine (PE)	1,2-dipalmitoyl-GPE (16:0/16:0)*
**4**	-0.017	0.003	<0.001	0.001	Unknown	Unknown	22162
**4**	0.019	0.004	<0.001	0.004	Unknown	Unknown	25837
**4**	-0.017	0.005	0.001	0.038	Unknown	Unknown	25950
**4**	0.012	0.003	<0.001	0.015	Unknown	Unknown	25955
**5**	0.015	0.003	<0.001	0.004	Nucleotide	Purine Metabolism, Adenine containing	1-methyladenine
**5**	0.037	0.009	<0.001	0.005	Amino Acid	Polyamine Metabolism	N-acetylputrescine
**5**	0.044	0.01	<0.001	0.005	Amino Acid	Phenylalanine Metabolism	phenethylamine
**5**	0.073	0.02	0.001	0.032	Energy	TCA Cycle	tricarballylate
**5**	0.026	0.007	<0.001	0.019	Amino Acid	Lysine Metabolism	N6-acetyllysine
**5**	0.018	0.004	<0.001	0.001	Amino Acid	Urea cycle; Arginine and Proline Metabolism	dimethylarginine (SDMA + ADMA)
**5**	0.027	0.007	<0.001	0.025	Amino Acid	Tyrosine Metabolism	tyramine O-sulfate
**5**	0.03	0.007	<0.001	0.004	Peptide	Dipeptide	cyclo(pro-sulfo-tyr)*
**5**	0.02	0.006	0.001	0.044	Amino Acid	Histidine Metabolism	1-methyl-5-imidazolelactate
**5**	0.024	0.004	<0.001	<0.001	Lipid	Primary Bile Acid Metabolism	chenodeoxycholic acid sulfate (2)
**5**	0.012	0.003	<0.001	0.014	Unknown	Unknown	12708
**5**	0.011	0.003	0.001	0.049	Unknown	Unknown	13553
**5**	0.022	0.005	<0.001	0.014	Unknown	Unknown	13729
**5**	0.032	0.007	<0.001	0.005	Unknown	Unknown	19220
**5**	0.026	0.006	<0.001	0.005	Unknown	Unknown	19917
**5**	0.027	0.006	<0.001	0.003	Unknown	Unknown	19921
**5**	0.027	0.007	0.001	0.031	Unknown	Unknown	19928
**5**	0.088	0.026	0.001	0.043	Unknown	Unknown	23662
**5**	0.026	0.008	0.001	0.037	Unknown	Unknown	23908

**5**	0.026	0.007	<0.001	0.03	Unknown	Unknown	24474
**5**	0.057	0.013	<0.001	0.004	Unknown	Unknown	25053
**5**	0.017	0.004	<0.001	0.019	Unknown	Unknown	25823
**5**	0.021	0.005	<0.001	0.014	Unknown	Unknown	25830
**5**	0.096	0.024	<0.001	0.013	Unknown	Unknown	25832
**5**	0.013	0.003	<0.001	0.014	Unknown	Unknown	25853
